# Trinucleotide repeat expansion length as a predictor of the clinical progression of Fuchs’ Endothelial Corneal Dystrophy

**DOI:** 10.1371/journal.pone.0210996

**Published:** 2019-01-25

**Authors:** Yu Qiang Soh, Gary Peh Swee Lim, Hla Myint Htoon, Xin Gong, V. Vinod Mootha, Eranga Nishanthie Vithana, Viridiana Kocaba, Jodhbir Singh Mehta

**Affiliations:** 1 Tissue Engineering and Stem Cell Group, Singapore Eye Research Institute, Singapore, Singapore; 2 Singapore National Eye Centre, Singapore, Singapore; 3 Ophthalmology Academic Clinical Program, Duke-NUS Graduate Medical School, Singapore, Singapore; 4 Singapore Eye Research Institute, Singapore, Singapore; 5 Department of Ophthalmology, University of Texas Southwestern Medical Center, Dallas, United States of America; 6 McDermott Center for Human Growth and Development, University of Texas Southwestern Medical Center, Dallas, United States of America; 7 Ocular Genetics Research Group, Singapore Eye Research Institute, Singapore, Singapore; 8 Department of Clinical Sciences, Duke-NUS Graduate Medical School, Singapore, Singapore; 9 Department of Ophthalmology, Yong Loo Lin School of Medicine, National University of Singapore, Singapore, Singapore; University of Florida, UNITED STATES

## Abstract

**Purpose:**

To determine if CTG18.1 TNR expansion length prognosticates the clinical progression of Fuchs’ Endothelial Corneal Dystrophy (FECD).

**Methods:**

This was a prospective cohort study. A total of 51 patients with newly diagnosed FECD were recruited and followed-up over a period of 12 years, from November 2004 to April 2016. Baseline clinical measurements included central corneal thickness (CCT), endothelial cell density (ECD) and CTG18.1 TNR expansion length from peripheral leukocytes, with yearly repeat measurements of CCT and ECD. A patient was defined to have experienced significant clinical progression and to have developed Threshold Disease if any of these criteria were fulfilled in either eye: a) CCT increased to >700μm, b) ECD decreased to <700 cells/mm^2^, or c) underwent keratoplasty for treatment of FECD.

**Results:**

Patients were categorized as having at least one allele whose maximum allele length was equal to or greater than 40 repeats (L≥40, n = 22, 43.1%), or having both alleles shorter than 40 repeats (L<40). Threshold Disease rates at the 5-year time point were 87.5% for the L≥40 group and 47.8% for the L<40 group (p = 0.012). This difference narrowed and was no longer statistically significant at the 8-years (92.9% vs 78.9%, p = 0.278) and 10-years (92.9% vs 84.2%, p = 0.426) time points.

**Conclusions:**

L≥40 patients are at greater risk of FECD progression and development of Threshold Disease within the first 5 years following diagnosis.

## Introduction

Fuchs’ endothelial corneal dystrophy (FECD) is one of the leading indications for endothelial keratoplasties (EK) in developed nations. In 2015, FECD accounted for 47.1% of all endothelial keratoplasty procedures performed in the United States of America.[1] FECD is characterized by guttate excrescences on the Descemet’s membrane (DM) located mostly within the central inter-palpebral zone, with relative sparing of the peripheries.[2] DM guttata are associated with dysfunctional corneal endothelial cells exhibiting morphological features such as pleomorphism and polymegathism, and derangements in the endothelial cell pump function.[3]

Quality of vision in patients with FECD may be compromised by corneal edema secondary to gradual failure of the corneal endothelial pump, and by guttae-induced visual disturbances such as decreased contrast sensitivity[4] and higher order aberrations.[5] Patients with early disease are usually amenable to conservative management with topical medications such as hypertonic saline. Thereafter, clinicians rely on various indicators including visual acuity, central corneal thickness[6], endothelial cell density and guttae density[7] to determine disease progression over time. Keratoplasty may be offered to patients with endothelial decompensation, and also increasingly, patients who are symptomatic for guttae-related visual disturbances in the absence of overt clinical edema.[5,8]

Over the past decade, significant progress has been made in the field of FECD genetics. While earlier studies have demonstrated possible associations between the FECD phenotype and mutations in genes such as FCD 1/2/3/4, *SLC4A1*, and *TCF8*,[9] there were significant inter-population variability in the strength of these associations, thus limited generalizability of these inheritance models. In recent years, numerous studies have demonstrated the association between a CTG trinucleotide repeat expansion sequence in the 3^rd^ intron of *TCF4* within chromosome 18q21.1[10] (henceforth referred to as the ‘CTG18.1’ locus), and the FECD phenotype.[11–19] The prevalence of the CTG18.1 repeat expansion in FED has been estimated to be much higher than any of the aforementioned putative gene candidates, having been identified at a prevalence of approximately 79% in German patients[12], 69.7% in Caucasians (United States of America) [13], and 43.9% amongst Singaporean Chinese patients[17]. Given such high prevalence, and that a causative relationship probably exists between an expanded CTG18.1 allele and the pathogenesis of FECD,[20] further investigations are warranted to investigate the possible implications which CTG18.1 allele length may have on the clinical FECD progression.

*TCF4* encodes the E2-2 transcription factor, which serves a regulatory function in the expression of multiple other genes.[21] Similar to myotonic dystrophy which is characterized by an expanded CTG trinucleotide repeat sequence in the DMPK gene[22], it is believed that these repeat expansion sequences negatively affect cellular function via the formation of toxic RNA foci[20,23] which sequesters critical splicing factors. Additionally, polynucleotide repeat expansion diseases are often characterized by an expansion bias[24], which is associated with a worsening of clinical manifestations with age. In recent years, evidence has emerged to suggest a direct correlation between repeat length at the CTG18.1 locus and FECD disease severity.[13] However, the predictive value of CTG18.1 trinucleotide repeat expansion length on clinical progression of FECD has not been well characterized. In this study, we investigated the relationship between CTG18.1 trinucleotide repeat expansion length on rate of clinical progression of FECD progression.

## Methods

In this prospective cohort study, all newly diagnosed FECD patients presenting to the corneal department in the Singapore National Eye Centre (SNEC) for the first time were evaluated. FECD was diagnosed based on the classic clinical features of DM guttae and corneal edema, and evidence of endothelial dysfunction on specular microscopy, such as the presence of a reduced corneal endothelial cell density, pleomorphism and polymegathism.[25] Inclusion criteria was that of FECD with disease severity of at least Grade 4 on the Krachmer clinical grading scale.[7] Patients who had previously undergone any form of ocular surgery, including cataract extraction surgery, were excluded. All patients were recruited following informed consent, with explanation of the nature and possible consequences of the study. Baseline measurements of CTG18.1 repeat expansion length, central corneal thickness and endothelial cell density were obtained. This study was performed in accordance to the tenets of the Declaration of Helsinki, with ethical approval granted by the Singapore Health Services (Singhealth) Institutional Review Board (IRB).

### Genotyping

Genotyping of the CTG18.1 trinucleotide repeat polymorphism was performed in a manner similar to that described previously.[13] 10mls of venous blood was obtained from each patient upon enrolment into the study, and the Nucleon blood extraction kit (Amersham Biosciences) was used to extract genomic DNA from peripheral leukocytes. Both short tandem repeat (STR) analysis and triplet primed polymerase chain reaction assays (TP-PCR) were performed.[16,17,26] While STR analysis was performed for all subjects, TP-PCR was performed to confirm the presence of an expanded allele when STR analysis detected only one allele or failed to detect any alleles.[16] In accordance to a previous study of FECD patients in a local population, an expanded allele was defined as one harboring a trinucleotide repeat sequence with ≥ 40 CTG repeats.[17] Patients with at least one allele in the CTG18.1 locus whose length was equal to or greater than 40 repeats were labelled as ‘L≥40’, while those with both alleles having fewer than 40 repeats were labelled as ‘L<40’.

STR analysis was limited by its ability to provide exact quantification of CTG repeat frequency only for alleles with fewer than 100 CTG repeats; Southern blot analysis would have been necessary to ascertain the exact number of CTG repeats for alleles containing more than 100 repeats. However, in accordance to our study design of grouping patients into the dichotomized groups of L≥40 vs L<40, such a high level of precision in determining CTG repeat frequency was not required, thus Southern blot analysis was not performed.

### Central corneal thickness and endothelial cell density

Central cornea thickness (CCT) was measured with the ultrasound pachymeter (Sonogage Inc, OH, USA), while endothelial cell density at the center of the cornea (ECD) was measured with a non-contact specular microscope (Konan Medical Corp., Hyogo, Japan), using the Center Method. When only a small number of endothelial cells were visible, the Flex-Center method was used. Qualified ophthalmic medical technicians were employed by the Singapore National Eye Centre to acquire these measurements. The value of each CCT and ECD measurement was taken as the mean of three consecutive readings. All measurements were acquired between 10am to 3pm, to reduce confounding secondary to diurnal variations in CCT.[27,28] Pachymetry and ECD readings were obtained for all patients during the baseline visit and yearly thereafter.

### Clinical progression

All patients were reviewed at least once per year. A patient was defined to have experienced significant clinical progression from baseline thus achieving the status of ‘Threshold Disease’, if at the time of assessment, any one of these 3 criteria were fulfilled for either eye: a) undergone keratoplasty, b) CCT greater than 700μm, or c) central ECD lesser than 700 cells/mm^2^. Data from both eyes of each patient was considered for the determination of Threshold Disease status, except for eyes which underwent cataract extraction surgery during the course of follow-up–in these cases, determination of clinical progression for that patient was based on the fellow eye which has not undergone cataract extraction surgery. Exclusion of eyes which have undergone cataract extraction surgery during follow-up was deemed necessary, as cataract extraction surgery variably accelerates corneal endothelial cell loss and the progression of FECD.

### Statistical analysis

All statistical analyses were performed using the Statistical Package for the Social Sciences version 22.0 (SPSS, Inc., Chicago, IL, USA). In terms of descriptive statistics, for continuous variables, the mean and standard deviation were calculated; for categorical variables, frequency distribution and categorical percentages were calculated. 1-way analysis of variance and the Fisher exact test were used to compare means and categorical distributions respectively. P value of less than or equals to 0.05 was considered statistically significant.

## Results

### Population demographics

**Fig 1:**
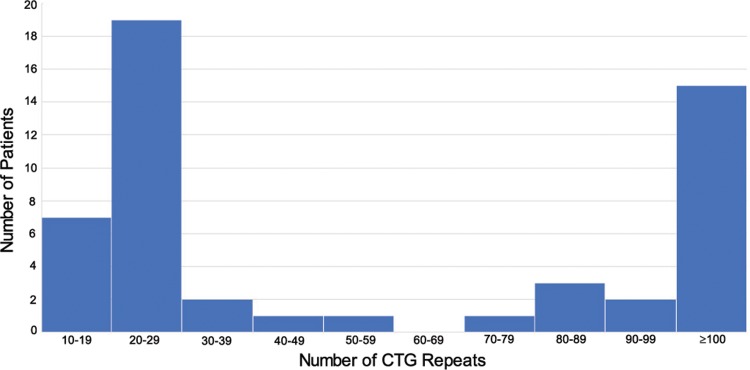
CTG Frequency distribution. Histogram representing the frequency distribution of CTG repeat length, when considering maximum CTG repeat length for each patient (i.e. number of CTG repeats in the longer allele).

Demographic data is summarized in Table 1. A total of 51 patients were recruited for this study, over a period of 12 years from November 2004 to April 2016. Patients were followed-up for an average of 4.32 years (range: 1–12 years). 67.2% (n = 32) were females, and 76.0% (n = 38) were of Chinese ethnicity (the rest being Indians, Malays, Eurasians and other non-local minority ethnicities). The average patient age was 60.6 ± 8.8 years old. The mean number of CTG repeats (in the *TCF4* locus) was 35.5 ± 30.9; 43.1% of patients (n = 22) had at least one allele which contained a trinucleotide repeat sequence of ≥ 40 repeats. A histogram representing the frequency distribution of maximum CTG repeat length is shown in Fig 1.

**Table 1:** Demographics.

	L≥40	L<40	P
**Number of patients**	43.1% (n = 22)	56.9% (n = 29)	-
**Age**	60.2 ± 7.78	61.0 ± 9.25	0.779
**% Female**	59.1% (n = 13)	65.5% (n = 19)	0.772
**% Chinese**	77.3% (n = 17)	72.4% (n = 21)	0.755
**Average number of CTG repeats**	57.1 ± 1.47	19.7 ± 0.66	< 0.001*
**Average baseline CCT (μm)**	611.4 ± 67.8	579.0 ± 58.8	0.022*
**Average baseline ECD (cells/mm**^**2**^**)**	1542 ± 635	1447 ± 733	0.618
**Duration of follow-up (years)**	4.00 ± 0.52	4.64 ± 0.50	0.385

All continuous variables (age, duration, number of repeats) are represented mean ± standard deviation. L≥40: At least one allele at the CTG18.1 locus with length greater than or equals to 40 CTG repeats; L<40: Both alleles at the CTG18.1 locus with lengths shorter than 40 CTG repeats.

*Statistically significant.

There were no statistically significant differences in age, gender and ethnicity distributions between the L≥40 and L<40 groups (Table 1). Average baseline CCT was greater in the L≥40 group compared to the L<40 group (611.4 ± 67.8 vs 579.0 ± 58.8 μm respectively, p = 0.022). There was no statistically significant difference in baseline central ECD between the L≥40 and L<40 group (1542 ± 635 vs 1447 ± 733 cells/mm^2^ respectively, p = 0.618). Both groups also had similar average follow-up durations of 4.00 ± 0.52 and 4.64 ± 0.50 years respectively (p = 0.385).

### Clinical progression

**Fig 2:**
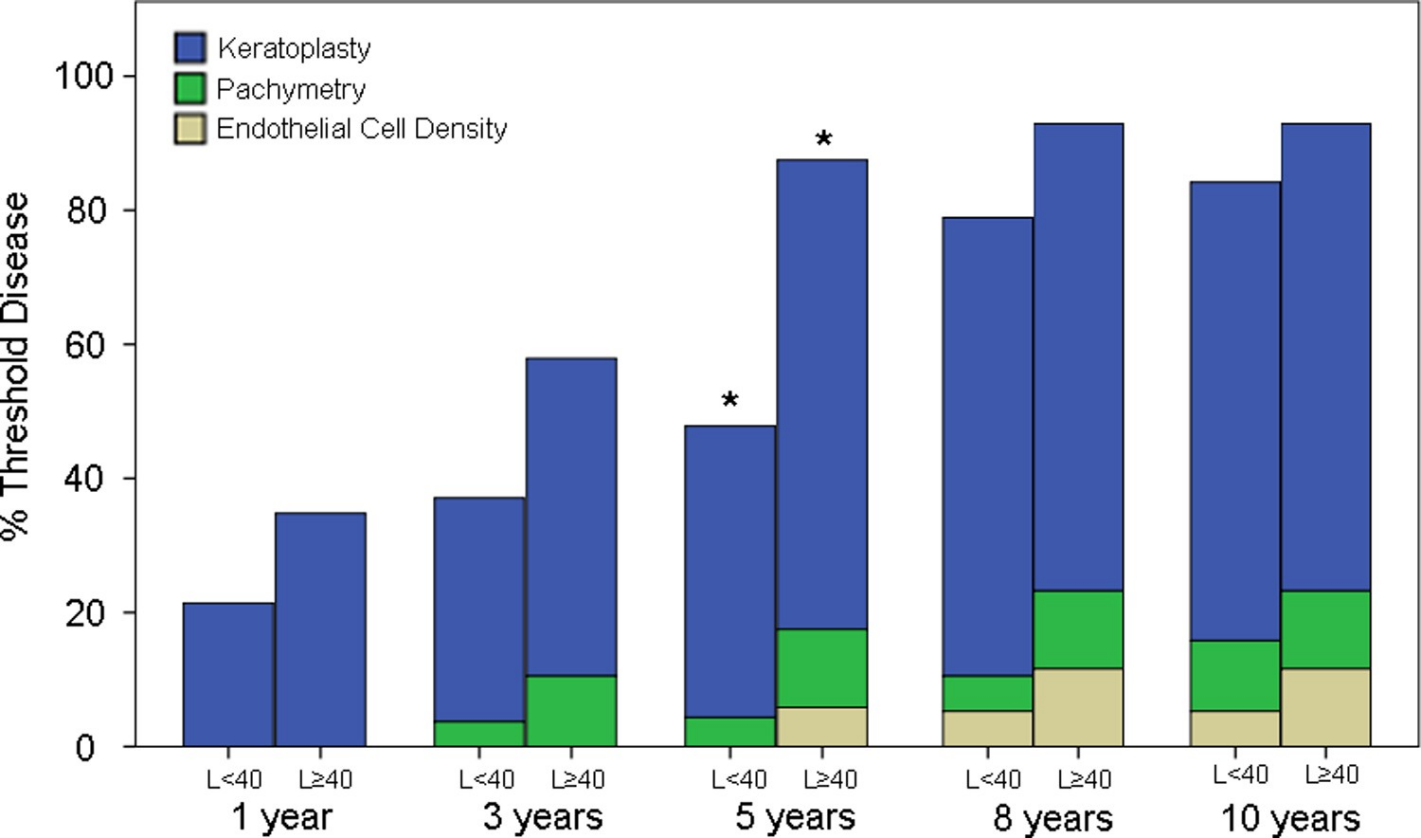
Threshold disease progression. Percentage of patients who had reached Threshold Disease, for L≥40 vs L<40 groups, by each time point. At each time point, patients are further sub-classified as having reached threshold based on the indication for keratoplasty, fulfilment of the pachymetry criteria or fulfilment of the endothelial cell density criteria. *Statistically significant difference in Threshold Disease proportions between L≥40 and L<40 groups, at 5-years (p = 0.012). There were no statistically significant differences in Threshold Disease proportions between the 2 groups at the 1-year (p = 0.227), 3-year (p = 0.065), 8-year (p = 0.278) or 10-year (p = 0.426) time-points.

Clinical information from a total of 51 and 45 patients were available at the 1-year and 3-year time points respectively. The number of patients with available clinical data declined to 39 at the 5-year time-point. Data from 33 patients were available at both the 8-year and 10-year time-points. The percentage of patients in the L≥40 vs L<40 groups who have experienced significant clinical progression and developed Threshold Disease by each time point is displayed in Fig 2. At 1-year, a slightly greater proportion of patients in the L≥40 group exhibited Threshold Disease compared to the L<40 group (34.8% vs 21.4%, p = 0.227). At 3-years, 57.9% of patients in the L≥40 group had progressed to develop Threshold Disease in contrast to 37.1% in the L<40 group, with the difference approaching statistical significance (p = 0.065). This disparity was accentuated at 5-years, with Threshold Disease rates of 87.5% vs 47.8% for the L≥40 vs L<40 groups respectively (p = 0.012). At 8-years, a significant proportion of patients in the L<40 group had also progressed to develop corneal decompensation, leading to a reduction of differences in Threshold Disease rates between then L<40 and L≥40 groups (92.9% vs 78.9% respectively, p = 0.278). This difference was further reduced at the 10-year time-point (92.9% vs 84.2%, p = 0.426), with the vast majority of patients in both groups having developed Threshold Disease. For both groups, at all time points, keratoplasty represented the main reason for patients satisfying the criteria of threshold disease.

**Fig 3:**
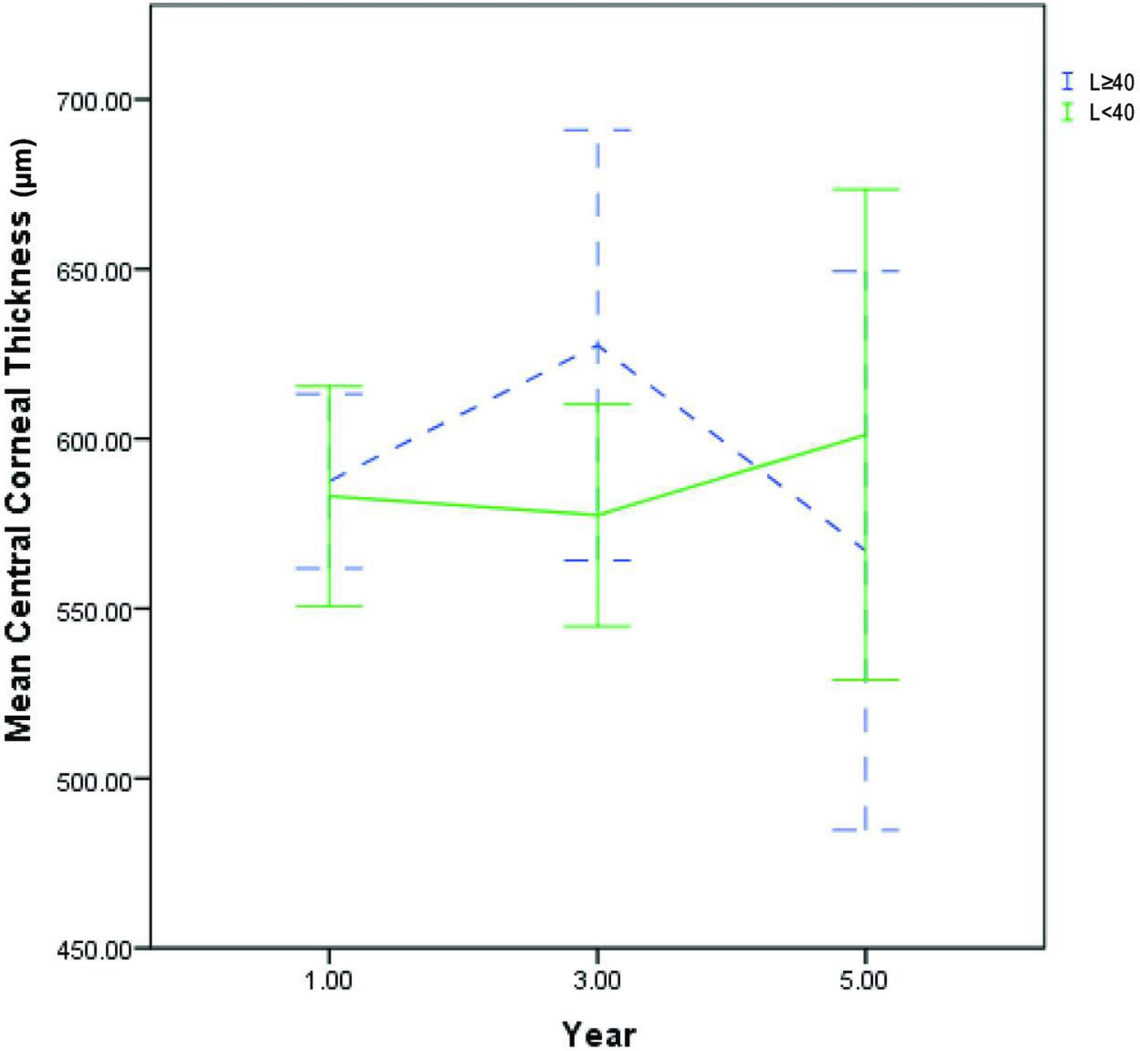
Central corneal thickness trend. Five-year trend of mean central corneal thickness for L≥40 (blue dotted line) vs L<40 groups (bold green line). Error bars represent 95% confidence intervals. There were no statistically significant differences between the L≥40 vs L<40 groups at all time points.

**Fig 4:**
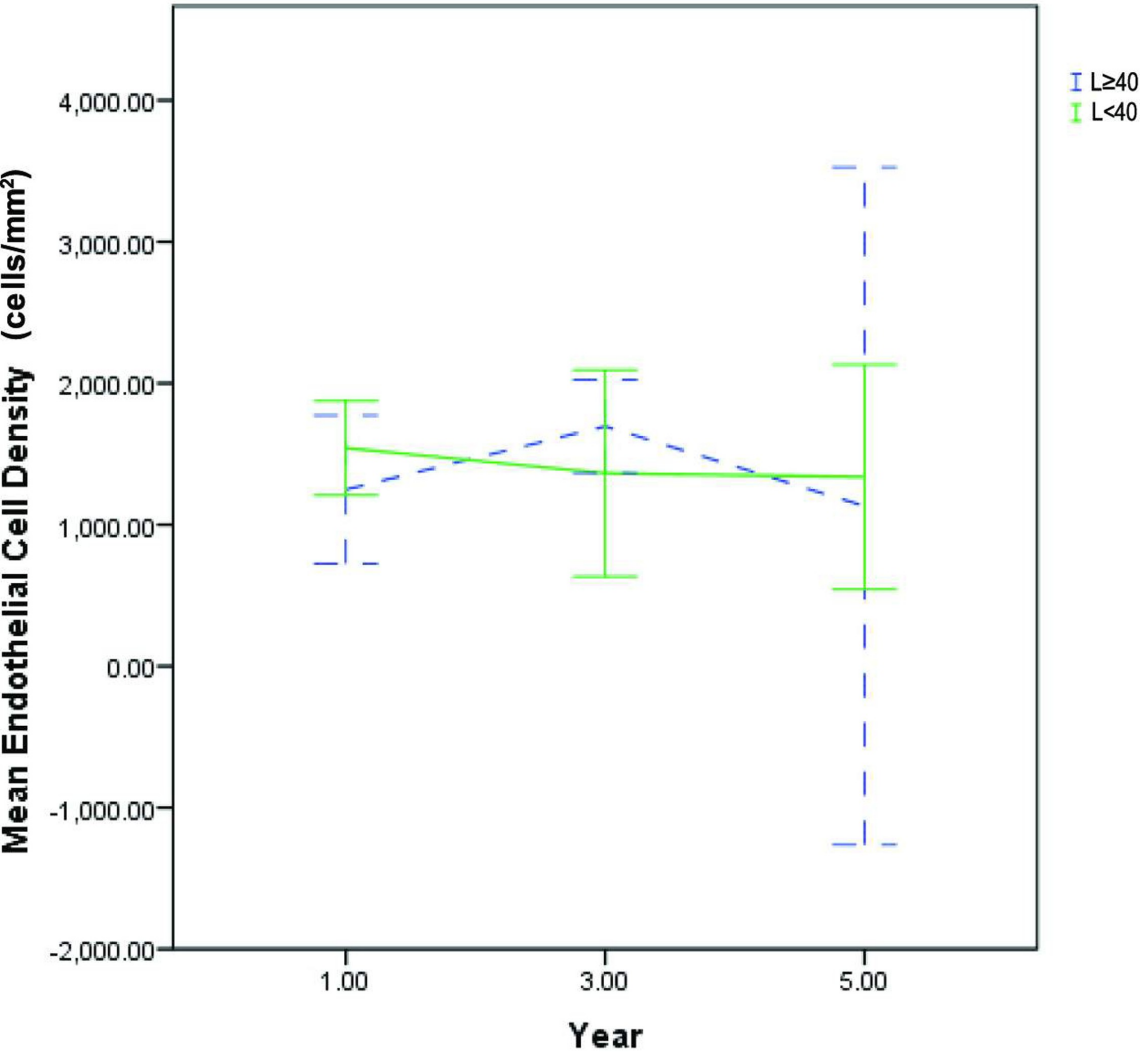
Endothelial cell density trend. Five-year trend of mean endothelial cell density for L≥40 (blue dotted line) vs L<40 groups (bold green line). Error bars represent 95% confidence intervals. There were no statistically significant differences between the L≥40 vs L<40 groups at all time points.

The mean CCT for the L≥40 vs L<40 groups were 587 ± 53 vs 583 ± 72 μm (p = 0.83) in the first year, 609 ± 64 vs 577 ± 68 μm (p = 0.188) in the third year and 581 ± 26 vs 601 ± 94 μm (p = 0.583) in the fifth year (Fig 3). The mean ECD for the L≥40 vs L<40 groups were 1249 ± 567 vs 1541 ± 495 cells/mm^2^ (p = 0.265) in the first year, 1694 ± 430 vs 1363 ± 789 (p = 0.3) in the third year and 1131 ± 266 vs 1337 ± 88 (p = 0.409) in the fifth year (Fig 4). There were no statistically significant differences in either CCT or ECD, between the L≥40 and L<40 groups, at all time points. There was scarce CCT and ECD data beyond the 5^th^ year of follow-up, as the majority of patients in the L≥40 group and a significant proportion of L<40 patients had already undergone keratoplasty by then. Additionally, the availability of ECD data beyond 5 years of follow-up was significantly limited by the number of patients who had progressed to develop significant corneal edema, which precluded accurate ECD measurements via specular microscopy, due to excessive light scatter.

## Discussion

Patients with early FECD who do not yet require keratoplasty are often managed conservatively with regular yearly follow-up evaluations. While the initial diagnosis and severity of FECD can be easily determined by a clinician during each visit, it may not be possible currently to accurately advise the patient on his or her risk of experiencing significant clinical progression potentially requiring keratoplasty in future. In this study, we found CTG repeat length to be a useful adjunct indicator which may be used to counsel an FECD patient regarding his or her risk of significant clinical progression and keratoplasty over the next 10 years. There was more rapid clinical progression of FECD amongst patients who harbor an expanded CTG18.1 allele (i.e. L≥40 group), during the first 5 years. This difference was subsequently reduced and eventually became statistically insignificant from 8 years onwards, due to clinical progression which also eventually occurred, albeit at a slower rate, amongst patients in the L<40 group. The observed phenomenon of a large majority of L<40 patients eventually progressing to develop threshold disease by the 8^th^ year is probably also reflective of the fact that FECD progression is dependent on genetic factors (i.e. CTG repeat expansion length) as well as epigenetics influences such as environmental exposure to oxidative stresses, which have not been characterized in this study.

In this study, we defined clinical progression, or ‘Threshold Disease’ based on several indicators including ECD decline, CCT increase or if the patient had undergone keratoplasty. While it may have been possible to evaluate the effect of genotype on FECD progression by selectively comparing the rates of visual acuity deterioration, ECD decline or CCT increase between the L≥40 and L<40 groups in isolation, we chose not to do so for several reasons. First, the incidence of visually-significant cataracts is high amongst patients in this study cohort whose average age was 60 years old, which precludes the use of visual acuity as a reliable gauge of FECD severity. While ECD quantification via specular microscopy is an objective indicator of FECD severity, it does not perform well and may in fact be impossible in some patients with severe central FECD, in whom significant light scatter by the oedematous cornea results in a low-quality view of the endothelial cell layer. While the Center method was employed for identification of endothelial cells, endothelial cells may still possibly be missed, especially in the ‘dark’ areas around guttata. Due to the abovementioned difficulties in accurately quantifying endothelial cell density especially in advanced disease, there is great variation regarding the density threshold beyond which FECD would be considered severe. As such, the use of < 700 cells/mm^2^ as threshold value in this study was based on our clinical experience of such a value being associated with relatively severe disease, rather than on any previously validated and standardized clinical threshold values, which in any case is unavailable.

CCT is probably the most reliable objective indicator of FECD, however CCT readings may fluctuate significantly due to diurnal variations in corneal hydration status.[28] Furthermore, there is also a natural variation in central corneal thickness even amongst clinically normal individuals.[29] For example, disease severity of an FECD patient whose current CCT is 580 μm but has a baseline thickness of 500 μm is significantly different from that of a patient whose baseline thickness was 560 μm. While the decision to undergo keratoplasty is a good indicator that disease progression had occurred to such an extent as to significantly impair visual function, it is dependent upon multiple other non-medical factors including but not limited to lifestyle requirements, cultural beliefs, access to care and financial circumstances. In order to improve the clinical applicability of our findings, it was decided that FECD progression would be best defined by a combined consideration of ECD, CCT and keratoplasty trend, instead of any one of those factors in isolation. The term ‘Threshold Disease’ was chosen because we felt that this set of criteria accurately represents the clinical threshold beyond which we would normally recommend keratoplasty to our patients. In contrast, a singular dependence on either CCT or ECD alone (Figs 3 and 4) would most likely not accurately reflect the rate of disease progression. Nonetheless, we would like to highlight that for both genetic groups at all time points, keratoplasty remains the main reason for patient satisfying the criteria of threshold disease. In contrast, a much smaller number of patients reached threshold disease by fulfilling the pachymetry and endothelial cell density criteria (Fig 2).

The findings of our study are in line with previously published work which indicated a greater risk of keratoplasty amongst patients who harbor an expanded CTG18.1 allele.[30] While the aforementioned was a cross-sectional study, this was a prospective cohort study which allowed quantification of the risk of disease progression over time. Besides looking at actual keratoplasty trends, we also included other objective indicators such as CCT and ECD in our definition of Threshold Disease, which we believe to contribute towards a more representative indicator of disease progression. While interpreting the results presented herein, it must be emphasized that this study was performed in a local Singaporean population, whose preferences and beliefs regarding keratoplasty may differ significantly from and thus may not necessarily be generalizable to other populations. For example, Singaporean patients are known to prefer to undergo keratoplasty only at a more advanced stage of the disease, when Snellen visual acuity has declined to an average of approximately 6/24,[31] in contrast to other populations, such as in the United States of America, where patients elect to undergo keratoplasty earlier, during which time Snellen visual acuity may be in the range of 6/15 or better.[32] The significant difference between the proportions of patients who have reached Threshold Disease (Fig 1) by each time point, vs the corresponding proportions of patients who had actually undergone keratoplasty (Fig 2), highlights the disparity between clinical indication and real-life patient choices.

As mentioned, there is now evidence to suggest that the CTG repeat expansion sequence is associated with accumulation of toxic intracellular RNA foci, which subsequently leads to corneal endothelial cell death and manifestation of the FECD phenotype.[20,23] In line with our observations of faster clinical disease progression in L≥40 patients, we hypothesize that L≥40 may possibly demonstrate a greater concentration of toxic intracellular RNA foci at the 5-year time point, compared to L<40 patients. While this would provide a mechanistic validation of our clinical observations, we were unable to make such a conclusion in this study as toxic intracellular RNA foci were not evaluated.

In this study, STR analysis of CTG repeat length was used to identify patients harboring alleles containing at least 40 CTG repeats, following by grouping of patients into L<40 and L≥40 categories. While we are aware that Southern blot analysis would have been necessary to precisely quantify CTG repeat frequency for alleles with more than 100 repeats, this was not performed as such a level of precision was not required for our statistical analyses. All statistical comparisons of disease progression were made between the dichotomized categories of L<40 vs L≥40; at no point in time was CTG repeat frequency regarded as a continuous variable against disease progression was evaluated. The only exception for which average allele length was treated as a continuous variable was when a comparison was made of the average allele lengths for the L<40 vs L≥40 groups (Table 1). In this case, alleles with >100 CTG repeats were assigned the default repeat length of 100, in order to facilitate estimation of mean allele length for each patient. Even with this approach, which represents the minimum possible mean allele length for patients with an allele comprising >100 CTG repeats, there was a statistically significant difference in mean allele length between the L<40 vs L≥40 groups (p<0.001).

The ages of patients recruited for this study varied within a significantly wide range of 60.6 ± 8.8 years old. As FECD is a progressive disease which is expected to worsen with age, it would have been ideal to assess the effect of CTG repeat expansion length on disease progression, while taking into account the effect of age. One way which we could have addressed this limitation would have been to only include patients belonging to a narrow age group (e.g. 55–60 years old). However, recruitment of patients would foreseeably be extremely slow with such a stringent inclusion criteria. The alternative would have been to statistically control for the effect of age when evaluating the correlation between CTG repeat length and disease progression, by including age as an independent predictive variable in a regression analysis. However, as expected a significant statistical correlation was found between age and CTG repeat length, thus precluding the inclusion of both age and CTG repeat length in such an analysis.

In summary, patients harboring an expanded CTG 18.1 allele are more likely to experience clinical progression of FECD during the first 5 years of life, compared to patients who do not carry the expanded allele. The inclusion of CTG 18.1 genotyping in the initial workup of FECD would allow clinicians to better advise their patients on their subsequent risks of progression and keratoplasty.
